# Multivariate analysis of immunohistochemical evaluation of protein expression in pancreatic ductal adenocarcinoma reveals prognostic significance for persistent Smad4 expression only

**DOI:** 10.1007/s13402-012-0072-x

**Published:** 2012-02-18

**Authors:** Niki A. Ottenhof, Folkert H. M. Morsink, Fiebo ten Kate, Cornelis J. F. van Noorden, G. Johan A. Offerhaus

**Affiliations:** 1 Department of Pathology, University Medical Center Utrecht, H04.312, Heidelberglaan 100, 3584CX Utrecht, The Netherlands; 2 Department of Pathology, Academic Medical Center, Amsterdam, The Netherlands; 3 Department of Celbiology and Histology, Academic Medical Center, Amsterdam, The Netherlands

**Keywords:** Pancreatic ductal adenocarcinoma, Immunohistochemistry, Prognosis, Long term survival, Smad4, Resection margin, Lymph node ratio

## Abstract

**Background:**

Pancreatic ductal adenocarcinoma (PDAC) has a dismal prognosis with a 5-year survival rate of <5% and an average survival of only 6 months. Although advances have been made in understanding the pathogenesis of PDAC in the last decades, overall survival has not changed. Various clinicopathological and immunohistological variables have been associated with survival time but the exact role that these variables play in relation to survival is not clear.

**Methods and results:**

To examine how the variables affected survival independently, multivariate analysis was conducted in a study group of 78 pancreatic ductal adenocarcinomas. The analysis included clinicopathological parameters and protein expression examined by immunohistochemistry of p53, Smad4, Axl, ALDH, MSH2, MSH6, MLH1 and PMS2. Lymph node ratio <0.2 (*p* = 0.004), tumor free resection margins (*p* = 0.044) and Smad4 expression (*p* = 0.004) were the only independent prognostic variables in the multivariate analysis. Expression of the other proteins examined was not significantly related to survival.

**Conclusions:**

Discrepancies with other studies in this regard are likely due to differences in quantification of immunohistochemical staining and the lack of multivariate analysis. It underscores the importance to standardize the methods used for the application of immunohistochemistry in prognostic studies.

## Introduction

Pancreatic ductal adenocarcinoma (PDAC) has a dismal prognosis with an annual mortality rate almost equaling incidence. Approximately 36.800 patients die annually from PDAC in the USA, making it the fourth leading cause of cancer-related death [1]. Five-year survival rates have not changed over the last decades and are currently still <5% [2–4]. Although advances have been made in the understanding of the pathogenesis of PDAC, these were not translated into improved prognosis [5]. Most patients present with locally advanced or distant metastatic disease, making resection with curative intent elusive. From the 5–20% of PDAC patients who qualify for resection only 10–18% will reach 5-year survival. Still, 5-year survival cannot be equated to cure because patients still die from recurrent disease after 5 years [6, 7].

Clinicopathological factors such as low disease stage [4, 8], resection margin [4, 8] and lymph node metastasis [9] have been associated with survival although the exact role of these characteristics in survival is unknown. Moreover, various tumor-specific protein expression patterns have been reported to be associated with overall survival in pancreatic cancer patients. Persistent expression of Smad4, a tumor suppressor gene affected in ~55% of PDACs, was found to be a strong prognosticator improving both disease-free and overall survival (OS) [10, 11]. Furthermore, micro-satellite instability, caused by defects in the mismatch repair (MMR) genes, has been reported to affect prognosis favorably [12] although not all studies confirm this [13, 14]. Another protein described to be associated with prognosis in PDAC is Axl, a receptor tyrosine kinase often involved in cancer development [15, 16]. Recently, a study by Rasheed et al. linked expression of aldehyde dehydrogenase (ALDH) to worse prognosis; it was suggested that ALDH-positive cells have tumor-initiating potential and that the percentage of ALDH-positive cells negatively affects OS [17]. However, in most of these studies, the clinical and histological characteristics were evaluated without adjustment for other variables that affect prognosis through a multivariate analysis.

To examine whether the different clinical and histological factors affect survival independently, a multivariate analysis was therefore conducted on a cohort consisting of 78 PDACs. The variables included clinicopathological parameters and expression patterns of most of the proteins previously reported to have a role in PDAC survival. The following proteins were examined: p53, Smad4, Axl, ALDH, and four mismatch repair genes; MSH2, MSH6, MLH1 and PMS2.

## Methods

### Patient selection

Paraffin-embedded tissue from 78 primary infiltrating PDACs was obtained from the Surgical Pathology archives of 3 cancer treatment centers: the University Medical Center Utrecht, the Academic Medical Center Amsterdam and the Erasmus Medical Center Rotterdam. Clinical data that was obtained included age, sex, tumor size, TNM-stage, histological grade, lymph-node status and exact survival time in months for all patients. Data were not available with respect to treatment of the patients or time of recurrence of the tumors and could therefore not be linked with the parameters investigated in this study.

### Tissue micro arrays (TMAs)

TMAs were developed using formalin fixed paraffin embedded tissue as previously described [8, 18]. Briefly, for each case representative areas containing neoplastic cells were marked on a hematoxylin-eosin stained sections which served as template. Three 0.6 mm cores were punched from the donor block and injected into the receiver block. For each patient, a 0.6 mm core from a non-neoplastic lymph node was included as control tissue.

### Immunohistochemistry (IHC)

IHC was performed on 4 μm-thick sections of the TMAs to analyse expression of p53, Smad4, Axl, ALDH and the four MMR proteins MSH2, MSH6, MLH1 and PMS2. Sections were deparafinized using routine techniques. Endogenous peroxidase activity was blocked with 3% H_2_O_2_ in methanol for 10 min after which sections were pretreated if necessary with ARS pH 9 for 10 min in the autoclave and cooled for 10 min. Before primary antibody application, sections were incubated with Protein Block Serum-Free (Dako Cytomation, Carpenteria, CA, USA). Then primary antibodies were applied. Antibody binding was visualized using the PowerVision + Poly-HRP kit (Immunologic, Duiven, The Netherlands) with 3,3-diaminobenzidin (DAB; Sigma-Aldrich, Seelze, Germany) or DAB + (Dako Cytomation) as chromogen. Sections were counterstained with hematoxylin and cover slips were applied. The primary antibodies that were used, their dilution and incubation time are described in Table 1.

**Table 1:** Antibodies used for immunohistochemistry

Antibody	Company	Pretreatment/Dilution	Incubation time	Substrate
p53 (BP53-12) Cat. #MS-738-7	Thermo Scientific, Fremont, CA, USA	ARS pH9/1:2000	1 h, room temperature	DAB
Smad4 (B-8) Cat. #sc-7966	Santa Cruz Biotechnology, Santa Cruz, CA, USA	ARS pH9/1:300	1 h, room temperature	DAB+
ALDH Cat. #61195	BD Transduction Laboratories, Franklin Lakes, NJ, USA	ARS pH9/1:200	1 h, room temperature	DAB
Axl Cat #AF154	R&D Systems, Minneapolis, MN, USA	None/1:100	1 h, room temperature	DAB
MLH1 Cat. #13271A	BD Pharmingen, San Diego, CA, USA	ARS pH9/1:50	Overnight, 4 C	DAB+
MSH2 Cat. # NA27	Oncogene Research Products, Schwalbach, Germany	ARS pH9/1:200	Overnight, 4 C	DAB+
MSH6 Cat. # 610919	BD Transduction Laboratories	ARS pH9/1:200	Overnight, 4 C	DAB+
PMS2 Cat. #556415	BD Transduction Laboratories	ARS pH9/1:500	Overnight, 4 C	DAB+

**Fig. 1:**
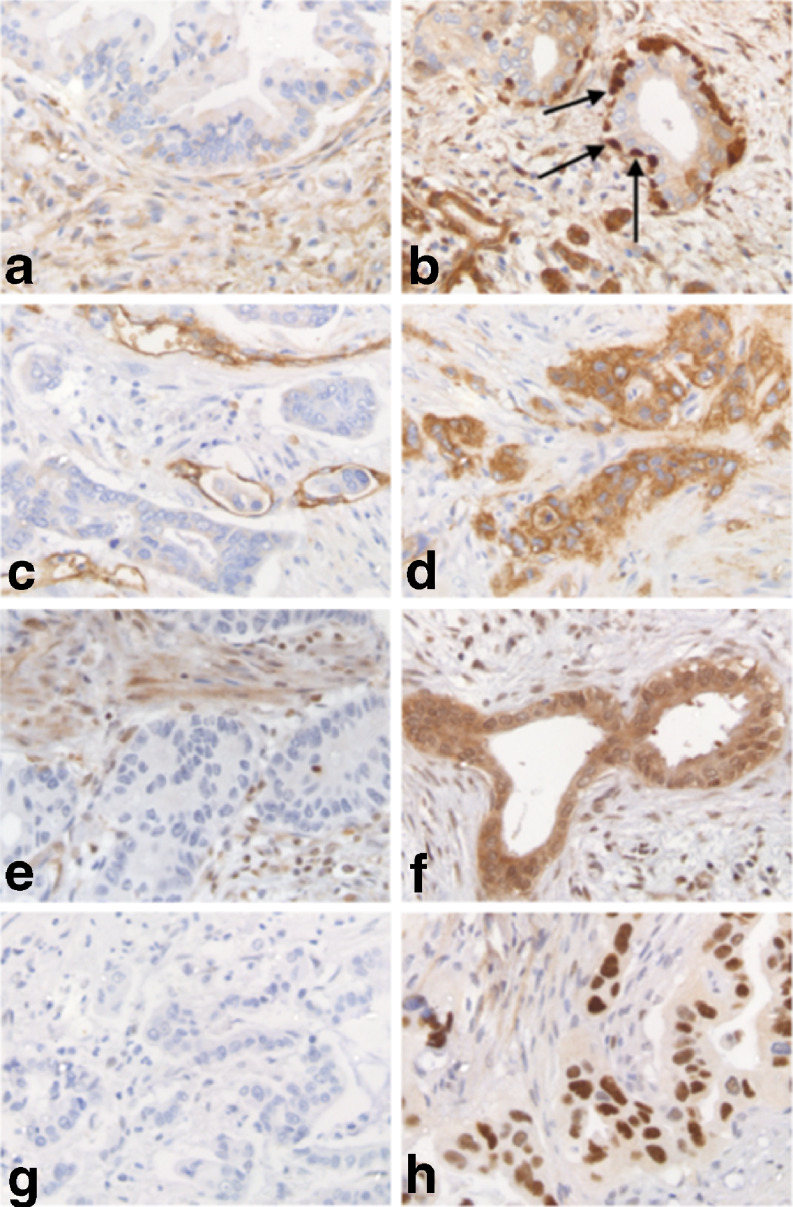
Immunohistochemical evaluation of both negative (**a,c,e,g**) and positive (**b,d,f,h**) expression of ALDH (**a**/**b**), Axl (**c**/**d**), Smad4 (**e**/**f**) and p53 (**g**/**h**). *Arrows* intense staining of ALDH in the basally located neoplastic cells

IHC labeling was scored by a single investigator after consensus was reached about cut off levels with an experienced pathologist behind a multiheaded microscope. In case of doubt, sections were again evaluated with the pathologist. Scoring was then based on consensus after discussion. The scoring method differed per antibody. P53 staining was scored as positive when nuclear accumulation of the protein was observed in more than 80% of the cancer cells. Absent or diffuse weak staining was scored as negative, indicating normal expression of p53. Smad4 and the MMR proteins were scored as negative when labeling was absent in the neoplastic cells. Weak or strong labeling was scored as positive. Axl staining was scored as negative when labeling was observed in 0–10% of the neoplastic cells. Slides showing labeling in >10% of the neoplastic cells were scored as positive. Scoring methods used in this study correspond with previously published papers [15, 19, 20]. For ALDH, scoring was performed in two different ways. First, sections were scored in a similar way as was described for Axl. The second way to score ALDH staining was according to the method reported by Rasheed et al. [17]. Only tumors exhibiting intense staining in both nuclei and cytoplasm of neoplastic cells, that was at least 2-fold higher than staining in normal pancreatic acinar cells adjacent to the tumor, were scored as positive (Figure 1). In most cases the 3 tissue cores showed similar staining intensity. In some cases, staining intensity differed between the cores. However, scoring was performed per tumor, not per core. Therefore, the 3 cores were evaluated as a whole and the IHC score was based on the total cancer cell population in the 3 cores.

### Statistical analysis

Statistical analysis was performed using SPSS version 15.0 (SPSS, Chicago, IL, USA). Associations between the different variables were examined using the Pearson’s Chi Square test. The survival was estimated by the Kaplan-Meier method and tested by log-rank test for statistical significance. Kaplan-Meier graphs were plotted using GraphPad Prism (GraphPad, La Jolla, CA USA). To evaluate correlations between the different variables, a Pearson’s correlation coefficient was calculated for both clinical characteristics and protein expression patterns. The variables with prognostic potential in the univariate analyses (*p* ≤ 0.10) and the variables that correlated significantly with one of the parameters were included in the multivariate analysis. Variables that did not correlate significantly were removed in a step-wise manner. The Cox proportional hazards model was used. A p-value <0.05 was considered to indicate statistical significance.

## Results

### Clinical characteristics

**Fig. 2:**
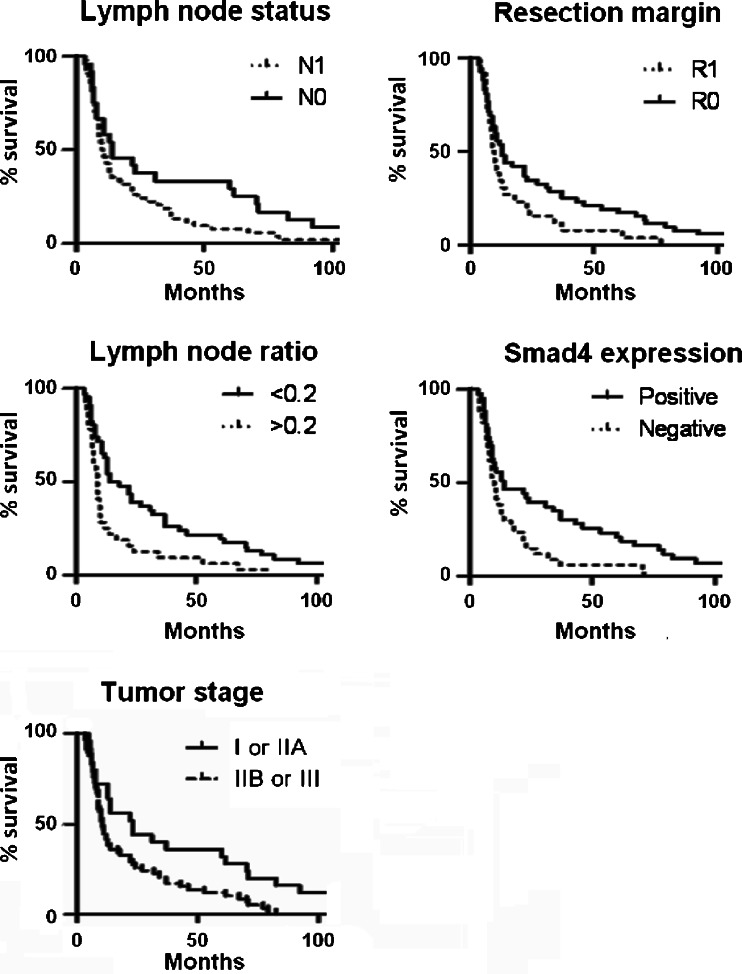
Kaplan-meier plots of overall survival of 78 PDAC patients in relation to node status (log-rank *p* = 0.066), resection margin (log-rank *p* = 0.056), lymph node ratio (log-rank *p* = 0.002), Smad4 expression (log-rank *p* = 0.008) and tumor Stage (log-rank *p* = 0.02)

The mean age of the 78 patients was 63 (range, 40–77). The diagnosis PDAC was confirmed in all patients. The median overall survival was 27 months. Twelve patients (15%) reached 5-year survival, 2 patients (3%) reached 10-year survival. Three patients presented with PDAC in the tail of the pancreas. The other 75 were diagnosed with a tumor in the head of the pancreas. Sixty-nine patients underwent a Whipple’s pancreatoduodenectomy. Seven other patients underwent either a pylorus-preserving pancreatoduodenectomy (*n* = 6) or a complete pancreatoduodenectomy (*n* = 1). Two patients underwent corpus/tail resection. Operation procedures did not affect survival (*p* = 0.48). Demographics and tumor characteristics are listed in Table 2. Univariate analysis revealed no differences in survival time related to age, gender, tumor size or histological grade. Absence of lymph node metastasis and a tumor-free resection margin showed a borderline significant correlation with improved survival (*p* = 0.07 and *p* = 0.06, respectively). The variables significantly improving survival as was shown by univariate analysis were lymph node ratio (LNR) (OS LNR < 0.2, 34.5 months; LNR > 0.2, 15.6 months; *p* = 0.002) and tumor stage (OS stage I or IIA, 43.1 months; stage IIB or III, 21.0 months; *p* = 0.02) (Figure 2).

**Table 2:** Distribution of demographic and tumor-related factors and univariate survival analysis for 78 PDAC patients

	Total (*n* = 78)	Median survival (months)	95% CI	p value (log-rank test)
Demographics				
Age (%)				0.69
< 65 years	39 (50%)	24.8	13.9–35.8	
≥ 65 years	39 (50%)	28.5	27.5–39.4	
Gender				0.76
male	35 (45%)	23.6	15.6–31.5	
female	43 (55%)	29.2	16.8–41.6	
Tumor characteristics				
Tumor size (cm) (1 case missing)				0.23
<2,0	13 (17%)	43.8	9.0–78.6	
≥2,0	64 (83%)	22.7	16.7–28.7	
Histological grade (1 case missing)				0.81
poor	21 (27%)	23.8	8.5–39.1	
moderate	40 (52%)	27.9	16.2–39.7	
well	16 (21%)	28.7	15.7–41.7	
Stage				0.02^a^
I or/IIA	20 (26%)	43.1	24.2–62.1	
IIB or III	58 (74%)	21.0	13.4–28.6	
Lymph node status (1 case missing)				0.07
N0	23 (43%)	38.7	21–56	
N1	54 (57%)	16.8	14–30	
Lymph node ratio (1 case missing)				0.002^a^
<0.2	46 (60%)	34.5	22.6–46.4	
≥0.2	31 (40%)	15.6	9.2–22.1	
Resection margin				0.06
R0	52 (67%)	31.6	22.6–46.4	
R1	26 (33%)	15.6	9.2–22.1	

^a^ Significant correlation with survival

### Immunohistochemical evaluation

We determined protein expression by immunohistochemistry for 8 different proteins. Strong nuclear staining of p53, indicating a defect in the protein, was found in 54% of the cases. Smad4 expression was completely absent in 43% of the cases. Staining of the mismatch repair proteins MSH-2, MSH-6, PMS-2 and MLH-1 was unaffected in 95%, 97%, 93% and 99%, of the cases, respectively. Axl expression was observed in 22% of the tumors. The other tumors were Axl negative. Although 75% of the tumors exhibited positive staining of ALDH protein, high intensity staining of ALDH protein as described by Rasheed et al.(17) was observed in only 11 tumors (16%).

Univariate analysis revealed Smad4 expression as a strong prognostic variable for survival (*p* = 0.008) (Table 3). The other variables did not have a statistically significant prognostic value.

**Table 3:** Univariate analysis for histological factors in 78 PDAC patients

	Total	Median survival	95% CI	p value (log-rank test)
Protein expression				
P53				0.84
negative	31 (46%)	30.1	14.1–46.0	
nuclear	36 (54%)	25.8	16.5–25.1	
Smad4				0.008^a^
negative	34 (44%)	15.8	10.4–21.2	
positive	49 (56%)	35.6	22.9–48.4	
Axl				0.40
negative	54 (78%)	23.6	15.6–31.5	
positive	15 (22%)	29.2	16.8–41.6	
ALDH (diffuse staining)				0.12
negative	18 (84%)	15.3	8.1–22.5	
positive	54 (16%)	28.6	19.1–38.1	
ALDH (high-expression)				0.11
negative	58 (84%)	22.7	16.7–28.7	
positive	11 (16%)	43.8	9.0–78.6	
MSH2				0.45
negative	4 (5%)	30.4	6.6–54.2	
positive	69 (95%)	25.15	16.2–39.7	
MSH6				0.73
negative	2 (3%)	35.2	0–86.6	
positive	70 (97%)	25.0	17.5–32.6	
MLH1				0.98
negative	1 (1%)	17.0	17.0–17.0	
positive	72 (99%)	25.7	18.3–33.2	
PMS2				0.41
negative	5 (7%)	15.2	3.9–26.6	
positive	70 (93%)	27.7	19.2–36.2	

^a^ Significant correlation with survival

### Multivariate analysis

To evaluate the correlation between the different variables, Pearson’s correlation coefficient was calculated for both the clinical characteristics and protein expression patterns. Tumor stage was significantly correlated with lymph node ratio (*p* < 0.001) and resection margin (*p* = 0.04). There was a strong correlation between expression of MMR proteins. MSH2 and MSH6 expression were strongly correlated (*p* = 0.005), as were PMS2 and MLH1 expression (*p* < 0.001).

The variables that had prognostic potential in the univariate analysis (*p* ≤ 0.10) or that were significantly correlated with one of the variables were subjected to multivariate analysis. The analysis included the variables lymph node status, LNR, resection margin, tumor stage and Smad4 expression. LNR <0.2, a tumor-free resection margin and persistent Smad4 expression significantly favored survival in multivariate analysis as shown in Table 4.

**Table 4:** Multivariate analysis including: tumor stage, lymph node status, lymph node ratio, resection margin, tumor stage and Smad4 expression

Variable		Hazard ratio	95% CI	p value
Tumor resection margin	R0	1.00		
	R1	1.86	1.02–3.41	0.044*
Lymph node ratio	N0	1.00		
	N1	2.36	1.37–4.07	0.002*
Smad4	negative	1.00		
	positive	2.34	1.30–4.21	0.004*

## Discussion

Although tremendous progress has been made over the last decades in the understanding of the pathogenesis of PDAC, PDAC patients still die within a few months after diagnosis. Critical analysis of factors involved in survival and prognosis potentially leads to a better understanding of the pathogenesis of PDAC. Therefore, identification of patient- and/or tumor-specific characteristics associated with increased survival time is an important strategy. Although multiple studies have been conducted concerning the prognostic significance of different variables, the picture remains incomplete and unclear. Tumor size, lymph node involvement, resection margin and histological grade have all been reported to significantly affect survival time (4, 8), although inconsistencies between studies remain [6, 21]. At present, only LNR was found to be a significant prognosticator in every study it was evaluated in [6, 9, 21, 22].

Apart from the different demographics, immunohistochemical evaluation of protein expression revealed multiple potential prognosticators in PDAC in recent publications [11, 15, 21–23]. However, multivariate analysis to evaluate whether these proteins have independent prognostic significance was generally not performed. In order to evaluate results from earlier publications on the prognostic relevance of clinical characteristics and to determine the role that various proteins play in prolonged survival, we performed multivariate analysis including most characteristics that have been implicated to be involved. Most importantly, we tried to identify which proteins remained significant for prognosis in multivariate analysis.

In the current study, LNR <0.2 was the strongest prognosticator which confirms results from other studies [23–25]. Another important prognostic factor that was described previously, persistent Smad4 expression [10, 11], was also a significantly favorable prognosticator with respect to survival in our cohort and remained so in multivariate analysis.

Positive Axl expression in PDAC has been related to shorter overall survival [15, 16]. Unfortunately, multivariate analysis was not performed by Koorstra et al. [15]. Song et al. [16] performed multivariate analysis including both Axl expression and lymph node involvement which resulted in a marginally significant effect of Axl on survival time. However, this study included only stage II PDACs making comparison with our results difficult. We did not detect a correlation between either Axl expression and lymph node status or Axl expression and survival time. Furthermore, only 23% of the tumors expressed Axl, which is a much lower proportion than described previously. It is possible that the scoring method for Axl expression in our study differed, leading to a smaller percentage of Axl-positive tumors. Because of these contradictory findings on Axl expression in PDAC, conclusions on the prognostic relevance of Axl expression cannot be drawn and it seems advisable to further delineate the role of Axl in PDAC development and progression.

Another group of proteins reported to affect survival are the MMR proteins MSH2, MSH6, MLH1 and PMS2. Mutations or epigenetic changes in these genes lead to microsatellite instability. Microsatellite instable tumors have been claimed to have a significantly better prognosis than their microsatellite stable counterparts [12, 13]. Although we did not assess microsatellite instability, we evaluated MMR protein activity using IHC. Similar to previously published data, approximately 13% of the tumors showed absence of expression of one or more of the MMR proteins [12, 26]. There was a strong correlation between the expression of the different mismatch repair proteins. MSH2 and MSH6 expression were strongly correlated, as were MLH1 and PMS2 expression. This was expected as both MSH2/MSH6 and MLH1/PMS2 form heterodimers through which they function in the repair of DNA mismatches [27]. However, we found no significant relationship between MMR protein expression and survival.

Recent reports on ALDH expression in malignancies focused on a small proportion of the cancer cell population (approximately 1% of the total tumor volume) which is characterized by a higher tumorigenic potential. These so-called ‘tumor-initiating cells’ have been under investigation in the last decade and a study on these ALDH-expressing ‘tumor-initiating cells’ in PDAC and the prognostic significance of their presence was recently published [17]. Although IHC revealed a large proportion of neoplastic cells exhibiting ALDH expression, Rasheed et al. [17] focused only on the cells that showed strong nuclear labeling of ALDH with a 2-fold or higher intensity as compared to the normal acinar cells. This resulted in a small percentage of ALDH-high tumors with significant prognostic potential. When scoring for expression of ALDH in our cohort, 75% of the tumors were positive, albeit with variable intensity. There was no relation between ALDH expression and survival time. When using the stringent scoring requirements as suggested by Rasheed et al. [17], the percentage of ALDH-high tumors was 16%. Again, we did not find a correlation between high ALDH expression tumors and survival time. Because cancer research is focused on ‘tumor-initiating cells’, it seems advisable to further investigate IHC staining of ALDH protein as our study demonstrates that discrepancies remain between the various studies.

IHC for the evaluation of protein expression is a fast and cheap method with great value in the laboratory. For example, IHC of Smad4 accurately mirrors Smad4 expression [28] and IHC demonstrates p53 defects as nuclear-bound protein. However, for most proteins there is not a standard method available for scoring IHC stained tumors and this makes interpretation and comparison of studies cumbersome. In the current study, this was illustrated in the ALDH expression analysis, where the two different scoring methods resulted in a different percentage of ALDH-positive tumors. Effort should be put into standardization of IHC scoring to increase the accuracy of evaluating protein expression using IHC to obtain valid quantitative data [29].

In conclusion, this study confirmed the prognostic significance of LNR and resection margin in PDAC in multivariate analysis. These two characteristics, together with Smad4 expression, had a significant effect on survival time and should be considered when determining patient specific prognosis. The expression patterns of the other proteins investigated had no significant relation with overall survival time in the multivariate analysis. Most of them have been reported to affect survival in other studies published, but more research has to be performed before a definitive conclusion can be drawn concerning the value of these proteins in determining prognosis.

## Abbreviations

PDAC: pancreatic ductal adenocarcinoma
MMR: miss-match repair
ALDH: aldehyde dehydrogenase
TMAs: tissue micro arrays
IHC: Immunohistochemistry
LNR: lymph node ratio
OS: overall survival
